# Texas to the Front

**Published:** 1902-09

**Authors:** 


### TEXAS TO THE FRONT.

**Our Epileptic Asylum.**

Dr. Worsham's paper in this issue, descriptive of the splendid asylum for epileptics now being constructed at Abilene under his supervision and nearing completion, will be read with great interest. In addition to the $200,000 appropriated for the purpose by the bill originally, the last Legislature gave $50,000 more, making a clean quarter million dollars for this splendid charity. Thus Texas sets the pace. This is far in excess of any appropriation made by any State for its epileptics. This act alone should make Governor Sayers's administration famous; but this is not all he has done for the unfortunates of Texas. By his advice such appropriations were made by the Legislature as to enable the State to double the capacity of its three lunatic asylums, and introduce all modern improvements for the safety, comfort and care of the inmates, and today, it is the proud boast of Texas that there is not an insane patient in any jail in Texas; they are all provided for by the State, in comfort, and under the best management. Appropriations for enlarging the asylums were made under the two administrations preceding that of Governor Sayers, but Culberson and Hogg promptly vetoed them—"cut them out." All honor to Governor Sayers.

